# Effects of cocoa extract supplementation and multivitamin/multimineral supplements on self-reported fractures in the Cocoa Supplement and Multivitamins Outcomes Study randomized clinical trial

**DOI:** 10.1093/jbmr/zjaf030

**Published:** 2025-02-18

**Authors:** Carolyn J Crandall, Sharon Chou, Eunjung Kim, Dana Ratnarajah, Nancy R Cook, Allison Clar, Howard D Sesso, JoAnn E Manson, Meryl S LeBoff

**Affiliations:** Division of General Internal Medicine and Health Services Research, Department of Medicine, David Geffen School of Medicine at University of California, Los Angeles, Los Angeles, CA 90024, United States; Division of Endocrinology, Diabetes and Hypertension, Brigham and Women’s Hospital, Boston, MA 02115, United States; Harvard Medical School, Boston, MA 02115, United States; Division of Preventive Medicine, Brigham and Women’s Hospital and Harvard Medical School, Boston, MA 02115, United States; Division of Endocrinology, Diabetes and Hypertension, Brigham and Women’s Hospital, Boston, MA 02115, United States; Harvard Medical School, Boston, MA 02115, United States; Division of Preventive Medicine, Brigham and Women’s Hospital and Harvard Medical School, Boston, MA 02115, United States; Department of Epidemiology, Harvard T.H. Chan School of Public Health, Boston, MA 02115, United States; Division of Preventive Medicine, Brigham and Women’s Hospital and Harvard Medical School, Boston, MA 02115, United States; Harvard Medical School, Boston, MA 02115, United States; Division of Preventive Medicine, Brigham and Women’s Hospital and Harvard Medical School, Boston, MA 02115, United States; Department of Epidemiology, Harvard T.H. Chan School of Public Health, Boston, MA 02115, United States; Harvard Medical School, Boston, MA 02115, United States; Division of Preventive Medicine, Brigham and Women’s Hospital and Harvard Medical School, Boston, MA 02115, United States; Department of Epidemiology, Harvard T.H. Chan School of Public Health, Boston, MA 02115, United States; Division of Endocrinology, Diabetes and Hypertension, Brigham and Women’s Hospital, Boston, MA 02115, United States; Harvard Medical School, Boston, MA 02115, United States

**Keywords:** cocoa, flavanol, fracture, osteoporosis, cosmos, multivitamin, clinical trial

## Abstract

Osteoporosis is a major public health problem among older adults. Forty percent of older US adults take multivitamin/multimineral (MVM) supplementation. The effects of MVM supplementation on fractures are unclear. Preclinical and observational studies suggest that MVM and flavanols may have beneficial effects on bone. We conducted an ancillary study to Cocoa Supplement and Multivitamin Outcomes Study (COSMOS; NCT05232669) designed to investigate incident fracture and injurious falls in 21 442 COSMOS participants (12 666 females aged ≥65 yr and 8776 males aged ≥60 yr) randomized in a 2 × 2 factorial design to 1 of 4 intervention groups: cocoa extract + MVM, cocoa extract + MVM placebo, cocoa extract placebo + MVM, or double placebo. The daily cocoa extract supplement contained 500 mg/d flavanols and 80 mg/d (–)-epicatechin (Mars Edge); the daily MVM supplement was Centrum Silver (Haleon). The median (interquartile range) duration of the intervention was 3.6 (3.2-4.2) yr. Annually, participants self-reported incident fractures. In intention-to-treat analyses, we examined the effects of cocoa extract and MVM on the primary outcomes of total clinical fracture (hip, upper leg, forearm/wrist, pelvis, upper arm/shoulder, spine, knee, or other), hip fracture, and nonvertebral fracture, and secondary outcomes of clinical spine, forearm/wrist, major osteoporotic, and pelvic fracture using Cox proportional hazards models. During the intervention period, 2083 incident clinical fractures occurred. Compared with placebo, cocoa extract was not significantly associated with lower risk of incident clinical fracture (adjusted hazard ratio [aHR] 1.03, 95% CI 0.95-1.12) or nonvertebral fracture (aHR 1.05, 95% CI 0.96-1.14). MVM supplementation was not associated with lower risk of total clinical fracture (aHR 1.09, 95% CI 1.00-1.19), hip fracture (aHR 1.06, 95% CI 0.80-1.42), or nonvertebral fracture (aHR 1.10, 95% CI 1.00-1.20). These findings do not support the use of cocoa extract or MVM to decrease fracture risk in older individuals not selected for pre-existing osteoporosis.

## Introduction

Osteoporosis affects up to 1 in 2 postmenopausal women and 1 in 5 older men,^1^ and more than 53 million adults in the United States.^2^ Therefore, interventions that decrease fractures are of great public health importance. While effective prescription medications exist to reduce fracture risk, there is interest in plant-based therapies to prevent fractures. Flavonoids are a class of polyphenols found in berries, tea, grapes, cocoa, and other plant-based foods.^3^ The flavanols, also known as flavan-3-ols, are a subgroup of flavonoids.^3^ Beneficial effects of flavanols on bone health have been demonstrated in previous studies in nonhuman animals. In vitro studies show that flavanols increase bone formation^4–13^ and decrease bone resorption.^12–24^ Similarly, in vivo studies have found that flavanol exposure increases bone formation,^25–27^ decreases bone resorption,^19^^,^^27^^,^^28^ improves or maintains bone structure on micro-computed tomography,^26^^,^^27^^,^^29^ and increases (or prevents the loss of) BMD,^26–29^ a strong determinant of fracture risk. A few observational studies in humans, based on food frequency questionnaires, have found positive associations between flavanol intake and BMD.^30^^,^^31^ However, to our knowledge, randomized clinical trials (RCTs) have not tested effects of cocoa flavanol supplementation on incident fractures in humans.

Cocoa is produced from the bean of the cacao tree *Theobroma cacao*^32^ and is a rich source of flavanols. The COcoa Supplement and Multivitamin Outcomes Study (COSMOS, #NCT02422745) is a randomized, double-blind, placebo-controlled, 2 × 2 factorial trial testing the effects of a cocoa extract supplement and a multivitamin/multimineral (MVM) supplement on cardiovascular disease and cancer in older persons (women aged ≥65 yr and men aged ≥60 yr).^33^ COSMOS provided an invaluable opportunity to examine whether cocoa flavanol extract supplement vs cocoa extract placebo decreases fracture risk among older persons. *COSMOS: Effects on Falls and Physical Performance* (National Institutes of Health R01 AG071611; NCT05232669) is an ancillary study to COSMOS designed to investigate effects of cocoa extract supplementation on risk of injurious falls resulting in healthcare utilization (primary aim), physical performance (secondary aim), and incident fractures (tertiary aim).

Because of the inclusion of MVM intervention groups in COSMOS, this ancillary study also provides an invaluable opportunity to examine whether MVM supplementation vs placebo decreases fracture risk among older persons. MVM supplements are the most common dietary supplements taken in the United States, with approximately one-third of adults^34^^,^^35^ (including 40% of older adults^36^^,^^37^) reporting regular MVM use. One might hypothesize that it is the vitamin D component that might dominate any protective effect of MVM on fracture risk. MVM supplements typically contain vitamin D, which is necessary for bone health (recommended daily allowances of 600 IU/d for adults <70 yr old and 800 IU/d for adults >70 yr old^38^). However, even an intake of 2000 IU/d of vitamin D, which exceeds the daily recommended allowance of vitamin D, was not associated with decreased fracture risk in the large VITamin D and omegA-3 (VITAL) RCT.^39^ Other components of MVM might be beneficial for bone health. Given the high prevalence of fractures and MVM use among older persons, even small beneficial effects of MVMs on fracture risk could have important public health implications. However, RCTs regarding the effects of daily MVM on fracture risk are lacking.^40^

We examined whether supplementation with daily cocoa extract supplementation, MVM supplementation, or the combination vs placebo for 3.6 yr reduces the risk of incident total clinical (hip, upper leg, forearm/wrist, pelvis, upper arm/shoulder, spine, knee, other), hip, and nonvertebral fractures among older US adults. Secondary outcomes included clinical spine, forearm/wrist, major osteoporotic (hip, spine, forearm/wrist, upper arm/shoulder), and pelvic fracture. We hypothesized that fracture risk would be lower in participants assigned to cocoa extract supplementation than in those assigned to placebo but did not expect lower fracture risk among persons assigned to MVM supplementation.

## Materials and methods

We used data from COSMOS, a randomized, double-blind, placebo-controlled, 2 × 2 factorial trial testing the effects of a cocoa extract supplement and a MVM supplement on cardiovascular disease and cancer.^33^ The COSMOS study design and participant characteristics have been previously described.^33^ COSMOS included 21 442 US women aged ≥65 yr recruited from active participants of the Women’s Health Initiative Extension Study and US males and females aged ≥60 yr recruited via mailings by Brigham and Women’s Hospital.^32^

Participants were excluded if they had a history of myocardial infarction, stroke, or a recent (within the past 2 yr) cancer diagnosis.^33^ Study recruitment occurred from June 2015 to March 2018, randomization occurred from April 2016 to March 2018, and the study intervention period ended 31 December 2020 (median treatment period [interquartile range] 3.6 [3.2-4.2] yr).^33^ The 21 442 COSMOS participants were randomized to 1 of 4 intervention groups: cocoa extract supplement+ MVM supplement (*n* = 5360), cocoa extract supplement+ MVM placebo (*n* = 5359), MVM supplement+ cocoa extract placebo (*n* = 5360), or double placebo (*n* = 5363).^32^ Placebo capsules were identical in appearance to the active tablets. At the end of the intervention period, 10 077 (94%) of 10 719 participants assigned to cocoa extract, 10 068 (94%) of 10 723 participants assigned to cocoa extract placebo, 10 087 (94%) of 10 720 participants assigned to MVM supplementation, and 10 058 (94%) of 10 722 participants assigned to MVM placebo were alive and actively participating in the study (Figure S1).^32^ The current analysis is based on data from all of those 21 442 participants who remained active study participants. Initial primary COSMOS trial results regarding cardiovascular disease and cancer outcomes were recently published.^32^^,^^41^ For the *COSMOS: Effects on Falls and Physical Performance* ancillary study, we used data from all 21 442 of the original COSMOS trial participants. Power calculations for this ancillary study were previously published.^42^

All participants provided written informed consent before enrollment in the parent COSMOS trial. The *COSMOS: Effects on Falls and Physical Performance* study is approved by the Human Research Committees and Institutional Review Board at Mass General Brigham. This report follows the CONSORT reporting guideline for clinical trials.

We obtained information regarding age, sex, race, ethnicity, medication use, dietary supplement use, health history, body weight, height, and physical activity level from baseline self-assessment questionnaires. BMI was calculated as body weight in kilograms divided by the square of height in meter. Participants were asked to rate their health as excellent, very good, good, fair, or poor.

Fracture prior to study randomization was assessed at baseline using the question “Other than a major accident such as a car accident or falling from a high ladder, have you ever broken any of these bones at age 50 and older? Mark all that apply.” Response choices included hip, spine, lower arm, upper arm, lower leg, upper leg, foot, and other bones. Information regarding osteoporosis-related medications, including alendronate, denosumab, ibandronate, raloxifene, teriparatide, zoledronic acid, risedronate, and calcitonin, was collected on baseline screening and annual questionnaires.

We calculated the Alternate Healthy Eating Index score (9 components each scored from 0 to 10 using each component’s specific cut-point, total score possible range 0-87.5), where a higher score indicates healthier diet.^43^^,^^44^ For this analysis, we divided the Alternate Healthy Eating Index score into tertiles, with the highest tertile indicating the healthiest eating pattern.^43^

In the parent COSMOS trial, at baseline, a subset of participants underwent measurement of an indicator of flavan-3-ol consumption, urinary 5-(3,4-dihydroxyphenyl)-γ-valerolactone-3/4-sulfate (gVL3S) and gVL-3/4-O-glucuronide metabolites (gVLM).^32^^,^^45^ Assays were performed using Ultra-Performance Liquid Chromatography–tandem mass spectrometry. Pre-randomization gVLM levels were available for 6508 participants of the current study.

### Interventions

The daily cocoa extra supplement contained a total of 500 mg/d of flavanols (including 80 mg/d (−)-epicatechin and 50 mg/d theobromine, provided by Mars Edge);^32^ the daily MVM supplement was Centrum Silver (provided by Pfizer Consumer Healthcare).^41^ The daily MVM supplement was Centrum Silver (provided by Haleon), containing calcium 220 mg/d, vitamin D 1000 IU/d, phosphorus 20 mg/d, and vitamin K 30 μg/d (full composition in Table S1).^41^

Compliance with study interventions (missing ≤8 d per mo of study pills) was greater than 80% at 12, 24, and 36 mo as well as at study closeout.^32^^,^^41^ Participants were not asked to limit dietary intake of cocoa products and were asked not to take their own personal MVM and cocoa supplements during the trial.

### Assessment of incident fractures

Fractures were self-reported by study participants. At the year one follow-up time point, on the main study self-report questionnaire, participants were asked, “In the past year, has a doctor or other health care provider told you that you had broken a bone?” Participants who answered this initial question affirmatively were then asked “Which bone(s)? Mark all that apply”. Response choices included hip, forearm/shoulder, spine, or other. Beginning in follow-up year 2, and annually thereafter, additional response categories were included: hip, upper leg (other than hip), forearm/wrist, pelvis, upper arm/shoulder, spine, or other. Participants were also asked to provide the date (mo/yr) when the fracture occurred.

We defined clinical fracture as fracture of the hip, upper leg, forearm/wrist, pelvis, upper arm/shoulder, spine, knee, or other. We defined nonvertebral clinical fracture as clinical fracture other than spine fracture. We defined major osteoporotic fracture as fracture of the hip, spine, forearm/wrist, or upper arm/shoulder.

### Statistical analysis

We separately examined the main effect of intention-to-treat with cocoa extract vs placebo and MVM vs placebo on incident fracture. We used Cox proportional hazards models to allow for variable follow-up time for each participant. The primary outcomes for this study were total clinical fracture, hip fracture, and nonvertebral fracture, as in our prior study evaluating fracture risk in relation to vitamin D supplementation vs placebo.^39^ We also prespecified clinical spine fracture, forearm/wrist fracture, major osteoporotic fracture, and pelvic fracture as exploratory outcomes. For this study, follow-up was censored at the date of first reported fracture, death, or end of trial intervention (31 December 2020), whichever came first.

We stratified the baseline hazard function by age, sex, study assignment to the alternate study intervention (MVM supplementation for the cocoa extract analyses, cocoa extract for the MVM analyses), and recruitment source (Brigham and Women’s Hospital or Women’s Health Initiative study).

We examined effect modification by prespecified baseline risk factors, consistent with our prior studies evaluating risk of fracture.^39^ In the cocoa extract vs placebo subgroup analyses, the risk factors were sex, age, race, BMI, AHEI, baseline chocolate intake, baseline urinary gVLM, history of diabetes, self-reported general health, randomization to MVM, use of prescription osteoporosis medication at baseline, and history of fragility fracture at baseline. In the MVM vs placebo subgroup analyses, the risk factors were sex, age, race, BMI, AHEI, history of diabetes, self-reported general health, randomization to cocoa extract supplementation, use of prescription osteoporosis medication at baseline, and history of fragility fracture at baseline. In the case of missing data in the stratified analysis, we recorded the number of participants with missing data for the variable. Data from participants with missing values for a given variable were excluded from the relevant subgroup analysis.

Statistical analyses were performed using SAS version 9.4 (SAS Institute). Statistical significance was defined as *p*-value <0.05 and/or 95% CI including the null value.

## Results

### Participant characteristics

Of the 21 442 participants, 12 666 (59.1%) self-identified as female and 8776 (40.9%) as male (Table 1). Mean (SD) age of participants at baseline was 72.1 (6.6) yr; 43.0% of participants were aged between 60 and 69 yr at baseline. Classified by self-reported race, there were 1131 African American/Black participants, 59 American Indian/Alaskan Native participants, 499 Asian/Pacific Islander participants, and 19 294 White participants. Ethnicity was reported as Hispanic/Latino by 544 participants. At baseline, the mean (SD) BMI of participants was 27.7 (5.4) kg/m.^2^ One-fifth of participants reported having experienced a fragility fracture (fracture not due to a major accident at age 50 or older) prior to study enrollment. The distribution of prevalent fractures according to fracture location is displayed in Table S4. At baseline, 6.7% of participants reported that they were taking prescription osteoporosis medication. There were no meaningful differences in other baseline characteristics of study participants assigned to receive supplemental cocoa extract compared to participants assigned to receive supplemental placebo (Table S2). The parent trial observed no significant effects of cocoa on nonmonitored cardiovascular, cancer, and other outcomes.^32^

**Table 1:** Baseline characteristics of participants in the overall COSMOS cohort (21 442), according to randomized treatment assignment.

Variable	All^a^	Cocoa extract	Cocoa extract placebo	MVM	MVM placebo
**Demographic characteristics**					
** Sex**	*N* = 21 442				
** Male, no. (%)**	8776 (40.9)	4382 (40.9)	4394 (41.0)	4382 (40.9)	4394 (41.0)
** Female, no. (%)**	12 666 (59.1)	6337 (59.1)	6329 (59.0)	6338 (59.1)	6328 (59.0)
** Age, mean (SD), yr**	72.08 (6.6)	72.08 (6.6)	72.07 (6.6)	72.1 (6.6)	72.1 (6.6)
** Race, no./total no.**	*N* = 21 442				
** Non-Hispanic White (%)**	19 294 (90.0)	9424 (89.8)	9670 (90.2)	9628 (89.8)	9666 (90.2)
** African American/Black (%)**	1131(5.3)	558 (5.2)	573 (5.3)	568 (5.3)	563 (5.3)
** American Indian/Alaskan Native (%)**	59 (0.3)	31 (0.3)	28 (0.3)	37 (0.4)	22 (0.2)
** Asian/Pacific Islander (%)**	499 (2.3)	274 (2.6)	225 (2.1)	258 (2.4)	241 (2.3)
** Multiracial/other/unknown (%)**	459 (2.1)	232 (2.2)	227 (2.1)	229 (2.1)	230 (2.2)
** Hispanic/Latino, no. (%)**	544 (2.6)	262 (2.5)	292 (2.8)	284 (2.8)	260 (2.5)
** BMI, mean (SD), kg/m^2^**	27.68 (5.4)	27.62 (5.3)	27.75 (5.5)	27.6 (5.4)	27.7 (5.5)
**Health history**					
** History of hip fracture in first-degree relative**	*N* = 20 153				
** Yes, no. (%)**	3530 (17.5)	1762 (17.4)	1768 (17.6)	1753 (17.5)	1777 (17.6)
** History of fragility fracture**	*N* = 21 442				
** Yes, no. (%)**	4246 (19.8)	2153 (20.1)	2093 (19.5)	2071 (19.3)	2175 (20.3)
** Had fall in the past year**	*N* = 21 321				
** Yes, no. (%)**	6844 (32.1)	3417 (32.1)	3427 (32.2)	3441 (32.3)	3403 (31.9)
** Leisure-time physical activity and stair climbing, total MET-hours/week, median (IQR)**	17.61 [5.7-33.5]	17.70 [5.6-33.8]	17.50 [5.8-33.4]	18.0 [5.9-33.7]	17.2 [5.6-33.4]
** Smoking**	*N* = 21 131				
** Current (%)**	835 (4.0)	398 (3.8)	437 (4.13)	417 (4.0)	418 (4.0)
** Past (%)**	8731 (41.3)	4396 (41.6)	4335 (41.0)	4345 (41.1)	4386 (41.5)
** Never (%)**	11 565 (54.7)	5766 (54.6)	5799 (54.9)	5808 (55.0)	5757 (54.5)
** Alcohol use**	*N* = 19 741				
** Daily (%)**	5276 (26.7)	2620 (26.6)	2656 (26.8)	2697 (27.3)	2579 (26.2)
** Weekly (%)**	7136 (36.2)	3600 (36.6)	3536 (35.7)	3557 (35.9)	3579 (36.4)
** Monthly (%)**	1462 (7.4)	725 (7.4)	737 (7.5)	751 (7.6)	711 (7.2)
** Rarely (%)**	5867 (29.7)	2897 (29.4)	2970 (30.0)	2891 (29.2)	2976 (30.2)
** Baseline use of supplemental cocoa extract**	*N* = 21 399				
** Yes (%)**	91 (0.4)	45 (0.4)	46 (0.4)	42 (0.4)	49 (0.5)
** Baseline use of multivitamin supplement(s)**	*N* = 21 359				
** Yes (%)**	8795 (41.2)	4438 (41.6)	4357 (40.8)	4413 (41.3)	4382 (41.0)
** Baseline use of supplemental vitamin D**	*N* = 21 166				
** ≤1000 IU/d, no. (%)**	8670 (41.0)	4351 (41.1)	4319 (40.8)	4331 (40.9)	4339 (41.0)
** >1000 IU/d, no. (%)**	4536 (21.4)	2302 (21.8)	2234 (21.1)	2238 (21.2)	2298 (21.7)
** Baseline use of supplemental calcium**	*N* = 21 183				
** ≤1200 mg/d, no. (%)**	9200 (43.4)	4621 (43.6)	4579 (43.2)	4596 (43.4)	4604 (43.5)
** >1200 mg/d, no. (%)**	1066 (5.0)	528 (5.0)	538 (5.1)	516 (4.9)	550 (5.2)
** Baseline chocolate intake**	*N* = 19 721				
** Daily, no. (%)**	2317 (11.8)	1134 (11.5)	1183 (12.0)	1154 (11.7)	1163 (11.8)
** Weekly, no. (%)**	11 129 (56.4)	5606 (57.0)	5523 (55.9)	5575 (56.3)	5554 (56.5)
** Monthly, no. (%)**	2923 (14.8)	1455 (14.8)	1468 (14.9)	1467 (14.8)	1456 (14.8)
** Rarely, no. (%)**	3352 (17.0)	1640 (16.7)	1712 (17.3)	1700 (17.2)	1652 (16.8)
** Baseline urinary gVLM, median^b^**	*N* = 6508				
** <Median (3.29) (%)**	3250 (49.9)	1640 (50.4)	1610 (49.5)	1579 (49.3)	1671 (50.5)
** ≥Median (3.29) (%)**	3258 (50.1)	1612 (49.6)	1646 (50.6)	1622 (50.7)	1636 (49.5)
** Alternative Healthy Eating Index**	*N* = 18 979				
** Lowest tertile, no. (%)**	6144 (32.4)	3031 (32.0)	3113 (32.8)	3060 (32.2)	3084 (32.6)
** Middle tertile, no. (%)**	6414 (33.8)	3237 (34.2)	3187 (33.4)	3176 (33.4)	3238 (34.2)
** Highest tertile, no. (%)**	6421 (33.8)	3209 (33.9)	3212 (33.8)	3275 (34.4)	3146 (33.2)

Abbreviations: gVLM, glucuronide metabolite; MVM, multivitamin/multimineral supplementation.

a Information was lacking regarding ethnicity in 863 participants, body mass index in 424 participants, and leisure time physical activity in 258 participants.

b 5-(3,4-dihydroxyphenyl)-γ-valerolactone-3/4-sulfate (gVL3S) and gVL-3/4-O-glucuronide metabolites (gVLM), a biomarker of flavanol intake.

There were no meaningful differences in baseline characteristics of study participants assigned to receive MVM supplementation compared to participants assigned to receive MVM placebo (Table 1 and Table S3). Forty-one percent of participants reported the use of MVM supplements at study baseline. MVM supplements were discontinued at study initiation.

### Associations between cocoa extra supplementation and incident fracture

During the study intervention period, 2083 incident clinical fractures were reported: 1056 in the cocoa extract intervention group and 1027 in the cocoa extract placebo group.

**Figure 1:**
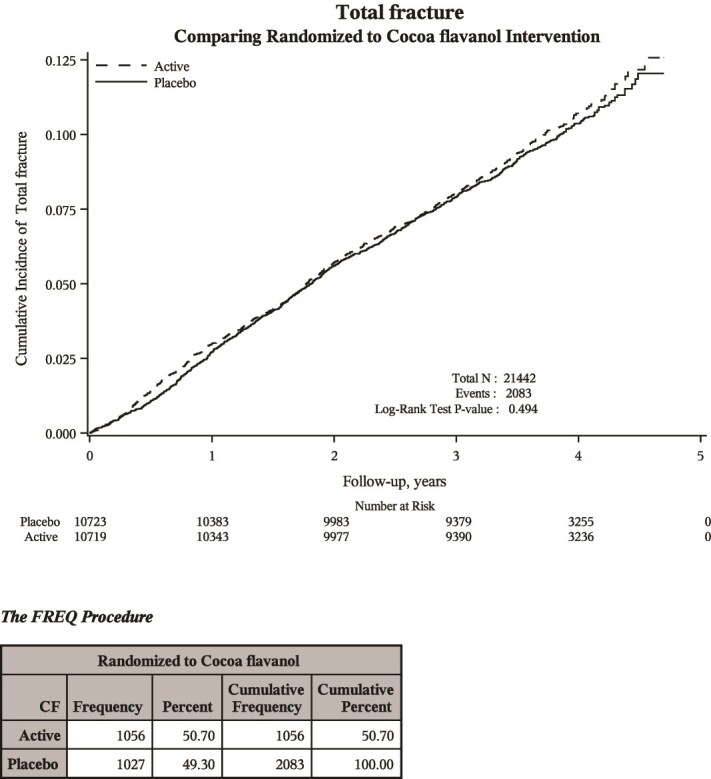
Kaplan–Meier curves displaying incident clinical fractures with cocoa extract vs placebo over time.

After stratification of the baseline hazard function by age, sex, study assignment to MVM supplementation, and recruitment source, cocoa extract (vs cocoa extract placebo) was not significantly associated with incident clinical fracture (Table 2 and Figure 1). This was true for each of the examined primary and secondary fracture outcomes: total clinical fracture (primary outcome), hip fracture (primary outcome), nonvertebral fracture (primary outcome), clinical spine fracture, forearm/wrist fracture, major osteoporotic fracture, and pelvic fracture.

**Table 2:** Effect of cocoa extract (vs placebo) on incident fracture in 21 442 COSMOS participants.

Fracture endpoint	All (*N* = 21 442)	Cocoa extract (*n* = 10 719)	Placebo (*n* = 10 723)	Hazard Ratio (95% CI)^a^	*p*-value
**Total clinical fracture^b^**	2083	1056	1027	1.03 (0.95-1.12)	.49
**Hip fracture**	184	88	96	0.92 (0.69-1.23)	.57
**Nonvertebral fracture^c^**	1949	995	954	1.05 (0.96-1.14)	.32
**Clinical spine fracture**	168	77	91	0.85 (0.63-1.15)	.28
**Forearm/wrist fracture**	330	171	159	1.08 (0.87-1.34)	.51
**Major osteoporotic fractures^d^**	877	431	446	0.96 (0.85-1.10)	.59
**Pelvic fracture**	95	47	48	0.98 (0.66-1.47)	.93

Abbreviation: COSMOS, Cocoa Supplement and Multivitamin Outcomes Study.

a Baseline hazard function stratified by age, sex, study assignment to multivitamin supplement, and recruitment source; hazard ratio adjusted for race/ethnicity.

b Total clinical fracture: fracture of the hip, upper leg, forearm/wrist, pelvis, upper arm/shoulder, spine, knee, or other.

c Nonvertebral fracture: clinical fracture as defined above but excluding spine fracture.

d Major osteoporotic fracture: fracture of the hip, spine, forearm/wrist, or upper arm/shoulder (year 2 and subsequently).

The effect of the cocoa extract supplement vs placebo on incident clinical fracture did not significantly vary by prespecified subgroups, including sex, age, race/ethnicity, BMI, baseline chocolate intake, history of diabetes, use of prescription osteoporosis medications at baseline, fragility fracture prior to study baseline, and baseline gVLM level (Table 3).

**Table 3:** Effect of cocoa extract supplementation (vs placebo) on incident total clinical fracture, in prespecified subgroup analyses.^a^

		Total clinical fracture^b^			
Subgroup	No. of participants	Cocoa extract	Placebo	Hazard ratio (95% CI)	*p*-value for interaction
		No. of participants with event			
**Sex**					.79
** Men**	8776	229	218	1.05 (0.88-1.27)	
** Women**	12 666	827	809	1.02 (0.93-1.13)	
**Age, yr**					.19
** 60-69**	9224	330	294	1.13 (0.96-1.32)	
** 70-79**	9525	510	509	1.00 (0.89-1.13)	
** ≥80**	2693	216	224	0.97 (0.81-1.17)	
**Race**					.60
** Non-Hispanic White**	18 887	968	950	1.03 (0.94-1.12)	
** Other**	2555	88	77	1.14 (0.84-1.55)	
**BMI, kg/m^2^**					.97
** <25**	7070	405	392	1.04 (0.90-1.19)	
** 25-<30**	8230	359	362	1.02 (0.88-1.18)	
** ≥30**	5718	256	241	1.05 (0.88-1.25)	
**Alternate Healthy Eating Index**					.20
** Highest tertile**	6421	334	326	1.03 (0.88-1.20)	
** Mid tertile**	6414	297	332	0.89 (0.76-1.04)	
** Lowest tertile**	6144	305	256	1.20 (1.02-1.42)	
**Baseline chocolate intake**					.33
** At least weekly**	13 446	662	654	1.01 (0.91-1.13)	
** Monthly or less**	6275	319	297	1.12 (0.95-1.31)	
**Baseline urinary gVLM^c^**					.87
** <Median (3.29)**	3250	174	161	1.07 (0.86-1.33)	
** ≥Median (3.29)**	3258	154	155	1.06 (0.84-1.32)	
**History of diabetes**					.57
** Yes**	2864	139	143	0.96 (0.76-1.21)	
** No**	18 569	917	883	1.04 (0.95-1.14)	
**General health, self-reported**					.09
** Excellent**	5633	256	206	1.22 (1.02-1.47)	
** Very good/good**	14 709	732	750	0.98 (0.89-1.09)	
** Fair/poor**	633	42	36	1.36 (0.86-2.16)	
**Randomization to multivitamin supplementation**					.41
** Active**	10 720	539	543	1.00 (0.88-1.12)	
** Placebo**	10 722	517	484	1.07 (0.95-1.21)	
**Use of prescription osteoporosis medication(s)**					.91
** Yes**	1413	115	109	1.03 (0.79-1.34)	
** No**	19 682	924	898	1.04 (0.95-1.14)	
**Baseline history of fragility fracture**					.17
** Yes**	4246	378	391	0.95 (0.82-1.09)	
** No**	17 196	678	636	1.07 (0.96-1.20)	

a Baseline hazard function was stratified the by age, sex, study assignment to multivitamin supplementation, and recruitment source.

b Defined as fracture of the hip, upper leg, forearm/wrist, pelvis, upper arm/shoulder, spine, knee, or other.

c 5-(3,4 -dihydroxyphenyl)-γ-valerolactone-3/4-sulfate (gVL3S) and gVL-3/4-O-glucuronide metabolites (gVLM), a biomarker of flavanol intake.

### Associations between MVM and incident fracture

During study follow-up, 1082 incident clinical fractures were reported in the MVM supplementation group, and 1001 incident clinical fractures were reported in the MVM placebo group.

**Figure 2:**
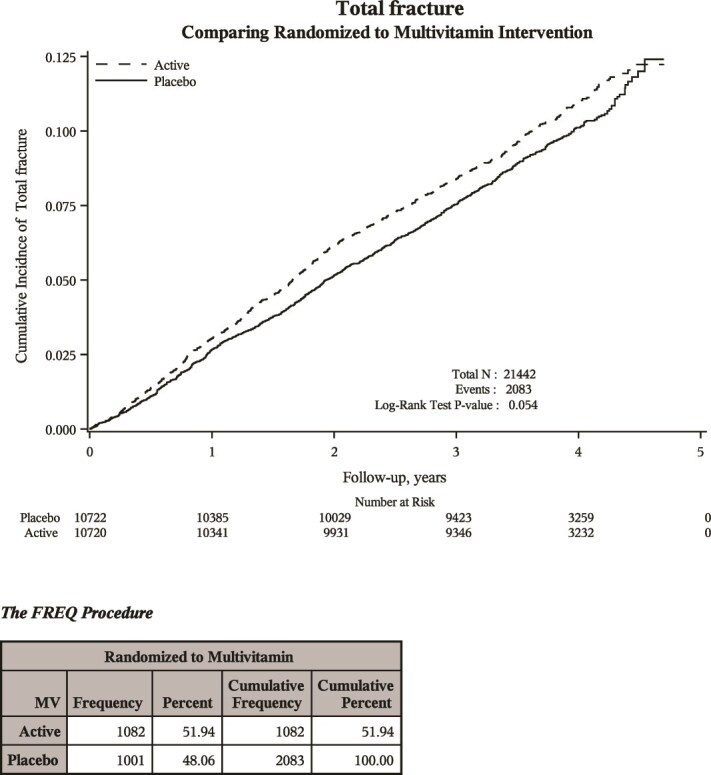
Kaplan–Meier curves displaying incident clinical fractures with multivitamin vs placebo over time.

In Cox proportional hazards regression models, the adjusted hazard ratio (aHR) for total clinical fracture was 1.09 (95% CI 1.00-1.19) and for nonvertebral fracture was 1.10 (95% CI 1.00-1.20) (Table 4 and Figure 2). There was no significant association between MVM supplement (vs MVM placebo) and incident hip fracture, clinical spine fracture, forearm/wrist fracture, major osteoporotic fracture, or pelvic fracture.

**Table 4:** Effect of multivitamin supplement (vs placebo) on incident fracture in 21 442 COSMOS participants.

Fracture incidence	All (*N* = 21 442)	Multivitamin supplement (*n* = 10 720)	Placebo (*n* = 10 722)	Hazard ratio (95% CI)^a^
**Total clinical fracture^b^**	2083	1082	1001	1.09 (1.00-1.19)
**Hip fracture**	184	95	89	1.06 (0.80-1.42)
**Nonvertebral fracture^c^**	1949	1015	934	1.10 (1.00-1.20)
**Clinical spine fracture**	168	92	76	1.21 (0.89-1.64)
**Forearm/wrist fracture**	330	168	162	1.04 (0.84-1.29)
**Major osteoporotic fractures^d^**	877	457	420	1.09 (0.95-1.24)
**Pelvic fracture**	95	41	54	0.76 (0.51-1.14)

Abbreviation: COSMOS, Cocoa Supplement and Multivitamin Outcomes Study.

a Baseline hazard function stratified by age, sex, study assignment to multivitamin supplement, and recruitment source; hazard ratio adjusted for race/ethnicity.

b Total clinical fracture: fracture of the hip, upper leg, forearm/wrist, pelvis, upper arm/shoulder, spine, knee, or other.

c Nonvertebral fracture: clinical fracture as defined above but excluding spine fracture.

d Major osteoporotic fracture: Fracture of the hip, spine, forearm/wrist, or upper arm/shoulder (year 2 and subsequently).

In a sensitivity analysis, after censoring of follow-up to end at the time of last contact, the magnitudes of associations were almost identical to results of the main analyses, with no significant associations between MVM supplementation (vs MVM placebo) and incident fracture (total clinical fracture aHR 1.09, 95% CI 1.00-1.19; nonvertebral fracture aHR 1.09, 95% CI 1.00-1.20).

In prespecified subgroup analyses, the magnitudes of associations of the MVM supplement vs placebo on incident clinical fracture appeared to vary by tertile of baseline AHEI score (Table 5). Among participants with the highest tertile of AHEI score, the aHR for clinical fracture was 1.27 (95% CI 1.09-1.48) but MVM (vs placebo) was not associated with clinical fracture risk for participants with lower tertiles of the AHEI score. However, none of the interaction effects met the criterion for significance after adjusting for multiple comparisons using the Bonferroni correction (ie, 10 subgroup tests resulting in an alpha value of 0.005 to define statistical significance).

**Table 5:** Effect of multivitamin supplementation (vs placebo) on incident total clinical fracture, in prespecified subgroup analyses.^a^

		Total clinical fracture^b^		
Subgroup	No. of participants	MVM	Placebo	Hazard ratio (95% CI)
		No. of participants with event		
**Sex**				
** Men**	8776	234	213	1.11 (0.92-1.33)
** Women**	12 666	848	788	1.09 (0.99-1.20)
**Age, yr**				
** 60-69**	9224	320	304	1.06 (0.91-1.24)
** 70-79**	9525	518	501	1.04 (0.92-1.17)
** ≥80**	2693	244	196	1.28 (1.06-1.55)
**Race**				
** Non-Hispanic White**	18 887	997	921	1.10 (1.00-1.20)
** Other**	2555	85	80	1.02 (0.75-1.39)
**BMI, kg/m^2^**				
** <25**	7070	432	365	1.19 (1.04-1.37)
** 25 to <30**	8230	368	353	1.04 (0.89-1.20)
** ≥30**	5718	247	250	1.04 (0.87-1.24)
**Alternate Healthy Eating Index score**				
** Lowest tertile**	6144	277	284	0.99 (0.84-1.17)
** Mid-tertile**	6414	314	315	1.02 (0.87-1.20)
** Highest tertile**	6421	373	287	1.27 (1.09-1.48)
**History of diabetes**				
** Yes**	2864	143	139	1.04 (0.82-1.32)
** No**	18 569	939	861	1.10 (1.00-1.20)
**General health, self-reported**				
** Excellent**	5633	225	237	0.95 (0.80-1.15)
** Very good/good**	14 709	784	698	1.13 (1.02-1.25)
** Fair/poor**	633	47	31	1.66 (1.04-2.65)
**Randomization to cocoa extract supplementation**				
** Active**	10 719	539	517	1.05 (0.93-1.19)
** Placebo**	10 723	543	484	1.13 (1.00-1.28)
**Baseline use of prescription osteoporosis therapy**				
** Yes**	1413	115	109	1.14 (0.87-1.49)
** No**	19 682	945	877	1.08 (0.98-1.18)
**History of fragility fracture at study baseline**				
** Yes**	4246	402	367	1.15 (0.99-1.32)
** No**	17 196	680	634	1.07 (0.96-1.19)

Abbreviation: MVM, multivitamin/multimineral.

a Baseline hazard function was stratified the by age, sex, study assignment to cocoa extract supplementation, and recruitment source. Prevalence of missing data was as follows: 2% for BMI, 12% for Alternate Health Eating Index, 0.04% for history of diabetes, 2% for general health, and 2% for baseline use of prescription osteoporosis therapy. There were no missing data for the other subgroups.

b Defined as fracture of the hip, upper leg, forearm/wrist, pelvis, upper arm/shoulder, spine, knee, or other.

The effects of MVM on incident fracture classified by fracture location were also examined according to AHEI score (Table S5). Although there was an initial pattern of higher magnitude of aHR values associated with MVM vs placebo among participants in the highest tertile (healthier eating pattern) of AHEI score at several fracture locations, none of these associations was significant after adjustment for multiple comparisons. A similar pattern of higher magnitude of fracture risk with MVM vs placebo among participants reporting fair/poor health than those with excellent or very good/good health, but again none of the associations was significant after adjustment for multiple comparisons (Table S6).

The effects of the MVM supplement on incident clinical fracture did not significantly vary by sex, age category, race, BMI, history of diabetes mellitus, randomization to cocoa extract intervention, use of prescription osteoporosis medication at baseline, or history of fragility fracture at baseline.

## Discussion

In this large RCT, cocoa extract supplementation for a median duration of 3.6 yr had no effect on incident clinical fracture risk among older persons. In prespecified subgroup analyses, the effects of the intervention did not differ by age, sex, BMI, or history of fragility fracture prior to randomization to treatment. In addition, MVM supplementation for a median duration of 3.6 yr was not associated with decreased risk of incident clinical fracture, hip fracture, or nonvertebral fracture among older persons. Also, no associations of MVM supplementation with forearm/wrist, major osteoporotic, or pelvic fracture were observed.

To our knowledge, no clinical trials have tested the effects of cocoa extract supplementation on fractures in humans. We had hypothesized that there would be an association between cocoa extract supplementation and decreased fracture risk because cross-sectional, observational, survey-based studies have found positive associations between dietary catechin intake^30^ or flavan-3-ol intake^31^ and BMD among older persons. In the study of Zhang and colleagues, the median (interquartile range) of the highest quartile of dietary flavan-3-ol intake was 353.3 (280.2-467.7) mg/d among women and 464.1 (355.9-476.1) mg/d among men. Our study supplement contained 500 mg/d of flavanols, which is higher than the highest quartile of intake reported in the Zhang study.

Animal studies also suggested potential benefits of cocoa extract supplementation on markers of osteoporosis. In vitro studies show that flavanols increase markers of bone formation^4–13^ and decrease markers of bone resorption.^12–24^ Similarly, in vivo studies in rodents have found that flavanol exposure increases markers of bone formation (eg, 5′ adenosine monophosphate-activated protein kinase, β-catenin, bone morphogenetic protein 2, trabecular number and volume, wingless-related integration site expression),^25–27^ decreases markers of bone resorption (eg, bone destruction, osteoclast formation, osteoclast surface and number, ovariectomy-induced increases in serum carboxy-terminal collagen crosslinks, urinary deoxy-pyridinoline),^19^^,^^27^^,^^28^ improves or maintains bone structure on micro-computed tomography,^26^^,^^27^^,^^29^ and increases (or prevents the loss of) BMD.^26–29^

It is useful to consider that 500 mg of flavanols contained in our cocoa extract intervention would be similar to the flavanol content of 90.7 grams (3.2 ounces) of dark chocolate or 771.1 grams (1.7 pounds) of milk chocolate. However, more than 3500 kcal/d of milk chocolate and nearly 500 kcal/d of dark chocolate would be required to obtain 500 mg/d of cocoa flavanols. Finally, chocolate is not a reliable source of cocoa flavanols, which can be destroyed in the harvesting of the cocoa beans and processing of the chocolate.

As is the case with clinical trials of cocoa extract, published results of RCTs regarding MVM supplementations’ effects on fracture risk are lacking. Previously published observational studies suggested that fracture risk would be lower among study participants assigned to receive MVM vs placebo. Beeram and colleagues’ systematic review on MVM and hip fracture (average participant age 69 yr, 21% male) concluded that MVM use was associated with significantly lower risk of fragility hip fracture (OR 0.49, 95% CI 0.32-0.77), but this estimate was only based on observational studies (6 case–control and 2 prospective cohort studies) because no RCTs met inclusion criteria (ie, hip fracture outcomes, English language, at least one-yr follow-up duration).^40^ Although it was not included in the meta-analysis of Beeram and colleagues, an RCT by Wang and colleagues involved 3318 participants (1461 men with mean age 55 yr and 1857 women with mean age 54 yr) in a nutritional intervention trial in Linxian, China (the Linxian dysplasia nutrition intervention trial, described in reference^46^). The RCT examined daily MVM (Centrum 2 tablets daily and one beta-carotene capsule daily) vs placebo for 6 yr, followed by 16-yr post-intervention follow-up.^47^ That trial reported finding “gender-specific effects”. Specifically, in men, the MVM supplement decreased risk of clinical fracture (spine, forearm, hip, femur, tibia, fibula fracture) by 63% during the trial period, which was not statistically significant (HR 0.37, 95% CI 0.10-1.39); however, the effect was statistically significant when analysis included both the trial period and the 5- or 10-yr post-intervention period (years 0-11, HR 0.38, 95% CI 0.15-0.97, *p* = .04; years 0-16, HR 0.46, 95% CI 0.24-0.89, *p* = .02; 1.2% loss to follow-up post-intervention).^47^ In contrast, in women, there was no significant effect of supplementation on fracture incidence, either during (HR 0.73, 95% CI 0.29-1.81) or after intervention. In COSMOS, effects of MVM on fracture did not significantly vary by sex.

The published report of Wang and colleagues states that “doses for most of the agents were two to three times higher than the USA recommended dietary allowances (RDAs), but ranged from 0.26 to seven times the RDA depending on the vitamin or mineral.”, that is, the previous RCT assigned 2, not one, Centrum MVM tablets daily (ie, 2 tablets, each containing 324 mg calcium phosphate and vitamin D 20 μg [800 IU]), along with an additional daily beta-carotene supplement. In contrast, the COSMOS RCT MVM intervention consisted of one Centrum Silver daily (including calcium 220 mg and vitamin D 1000 IU). However, we note that in the large VITamin D and omegA-3 TriaL (VITAL, *n* = 25 871 women aged 55 yr and older and men aged 50 yr and older), supplemental vitamin D at a dosage of 2000 IU/d had no effect on incident fractures.^39^ Therefore, it is unlikely that the vitamin D component of the Centrum Silver MVM is sufficient to reduce fracture risk.

Although MVMs have not been demonstrated to reduce fracture risk in RCTs, MVMs may have other health benefits. A meta-analysis of 4 RCTs performed for the United States Preventive Services Task Force concluded that MVM use was associated with a lower incidence of any cancer (odds ratio 0.93, 95% CI 0.87-0.99; absolute risk difference in RCTs −0.2% to −1.2%).^48^ Also, MVM was associated with benefits for episodic memory and global cognition in a meta-analysis of 3 separate placebo-controlled ancillary trials in COSMOS.^49^

This clinical trial has several strengths, including its randomized, double-blind, placebo-controlled design, large sample size with reasonable racial and ethnic diversity (1131 [5.3%] African American/Black participants, 59 [0.3%] American Indian/Alaskan Native, 499 [2.3%] Asian/Pacific Islander participants, and 544 [2.6%] Hispanic/Latino participants), inclusion of women and men, a long study duration, and high compliance with study interventions. Moreover, to our knowledge, this is the first large RCT of cocoa extract on incident fractures in humans and the first large RCT to test effects of MVM supplements on incident fractures among older persons living in the United States.

Potential limitations of this study include that fractures were self-reported. However, a previous validation study demonstrated that information obtained by self-report regarding fractures is good in the Women’s Health Initiative study (one of the 2 recruitment sources for the COSMOS trial). For example, fractures were confirmed by medical record review for 78% of self-reported hip fractures and 81% of self-reported forearm/wrist fractures.^50^ Validity was lowest for clinical spine fractures (51% confirmed by medical records), which was why clinical spine fracture was a secondary and not a primary endpoint. Additional information regarding validity of self-report comes from the VITAL trial, which had similar recruitment strategies and design to the COSMOS trial, and used the same question to ask about self-reported fractures.^39^ In that trial, 93% of self-reported fractures were confirmed on medical record review. Second, the *COSMOS: Effects on Falls and Physical Performance* study is an ancillary study, and fractures were not the major endpoint of the parent COSMOS trial. Information regarding BMD was obtained only in a small subset of participants (*n* = 493), and information was not available regarding bone turnover markers. It is possible that the dosage of cocoa extract and/or the duration of the intervention in COSMOS was inadequate to significantly influence fracture risk. Third, because COSMOS participants were community-dwelling older adults, our results may not be generalizable older adults living in institutions, nor to patients with pre-existing osteoporosis. COSMOS participants were not selected on fracture risk. Finally, it is possible that the timing of the cocoa intervention was too late relative to already established decrements in bone health of the older adult study population.

In conclusion, compared to placebo, neither cocoa extract supplementation nor MVM supplementation decreased risk of clinical fracture in generally healthy, community-dwelling older adults not selected for pre-existing osteoporosis during a median intervention period of 3.6 yr.

## Supplementary Material

supple_figure_1_zjaf030

Supplementary_Material_zjaf030

## Data Availability

The data set(s) will be deidentified prior to release for sharing. We will make the data and associated documentation available to users only under a data-sharing agreement. Details on the availability of the study data to other investigators will be on our study website at https://cosmostrial.org/.
