# Supplementary material for: Effects of cocoa extract supplementation and multivitamin/multimineral supplements on self-reported fractures in the Cocoa Supplement and Multivitamins Outcomes Study randomized clinical trial
Source: J Bone Miner Res. 2025 Feb 18;40(5):591–602. doi: 10.1093/jbmr/zjaf030 (PMC12103720; doi:10.1093/jbmr/zjaf030)
Supplement: supple_figure_1_zjaf030 [file supple_figure_1_zjaf030.pdf]

Supplemental Figure 1. Screening, Randomization, and Follow-Up of the Participants

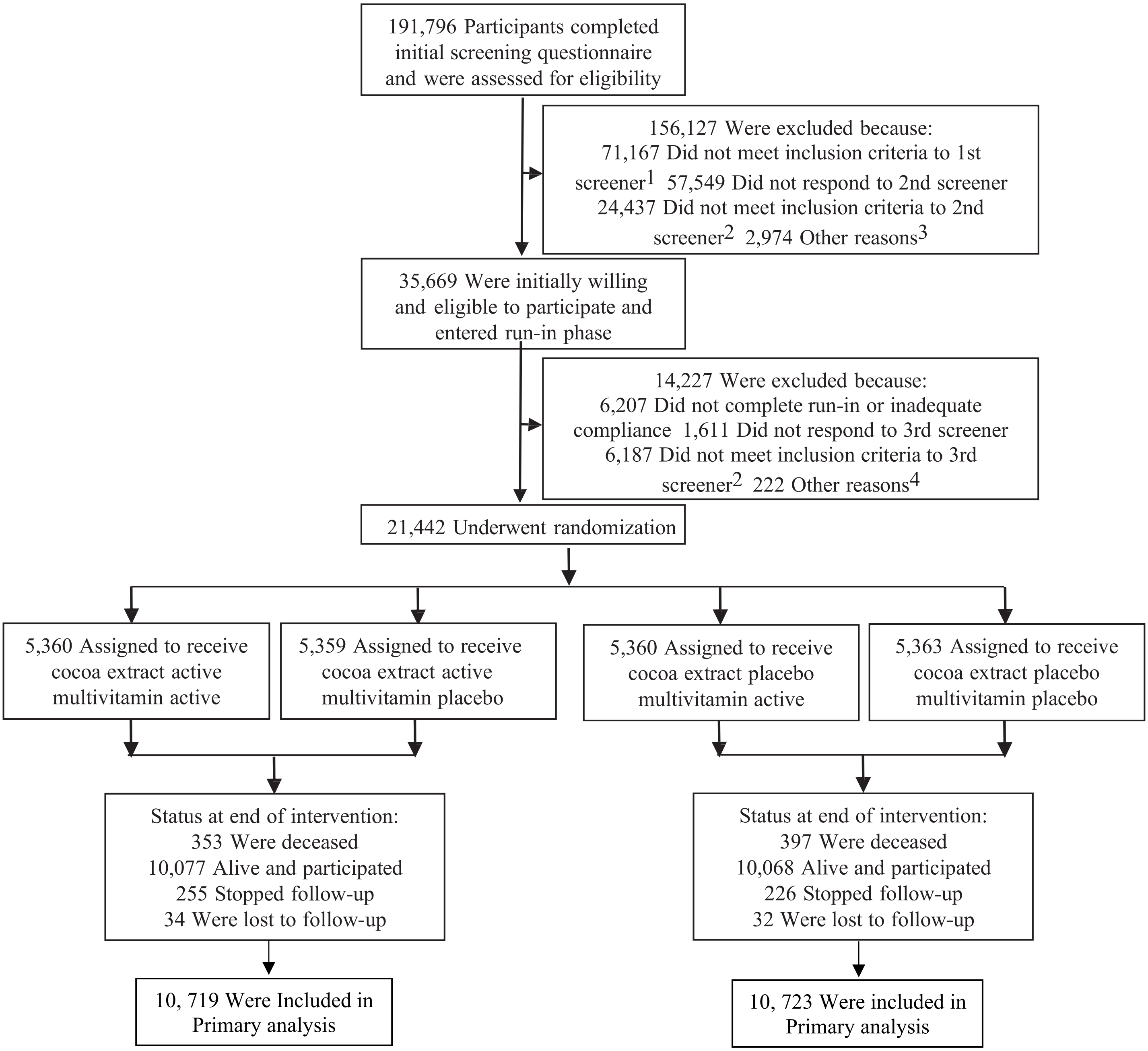

[1]Eligibility was determined by medical history, age, and willingness to forego personal use of cocoa extract and multivitamin pills. [2]Eligibility was determined by medical history, age, willingness to forego personal use of cocoa extract and multivitamin pills, caffeine sensitivity, and willingness to limit calcium and vitamin D supplement use. [3]Included subjects who never completed the screening phase (n = 2914), eligibility could not be determined (n = 5), and enrollment goal already met (n = 55). [4]Included subjects who never completed screening phase (n = 168) and enrollment goal already met (n = 54). *Figure reproduced with permission from the authors Sesso, H.D. et all, Am J Clin Nutr* 2022;115:1501–1510.
