# Supplementary material for: Effects of cocoa extract supplementation and multivitamin/multimineral supplements on self-reported fractures in the Cocoa Supplement and Multivitamins Outcomes Study randomized clinical trial
Source: J Bone Miner Res. 2025 Feb 18;40(5):591–602. doi: 10.1093/jbmr/zjaf030 (PMC12103720; doi:10.1093/jbmr/zjaf030)
Supplement: Supplementary_Material_zjaf030 [file supplementary_material_zjaf030.pdf]

Supplementary Material

Effects of Cocoa Extract Supplementation and Multivitamin/Multimineral Supplements on Self-Reported Fractures in the Cocoa Supplement and Multivitamins Outcomes Study (COSMOS) Randomized Clinical Trial

Carolyn J. Crandall, MD, MS, MACP, Eunjung Kim, MS, Sharon Chou, MD, Dana Ratnarajah, BS, Nancy R. Cook, ScD, Allison Clar, BS, Howard D. Sesso, ScD, MPH, JoAnn E. Manson, MD, DrPH, Meryl S. LeBoff, MD

**Item**	**Page number**
Supplemental Table 1. Composition of the COSMOS Centrum Silver Supplement	2
Supplemental Table 2. Baseline characteristics of participants in the overall COSMOS cohort (21,442), according to randomized treatment assignment: Cocoa Extract Supplementation vs. Placebo	3
Supplemental Table 3. Baseline characteristics of participants in the overall COSMOS cohort (21,442), according to randomized treatment assignment: Multivitimain/Multimineral Supplement vs. Placebo	6
Supplemental Table 4. Locations of Prevalent Fractures Prior to Trial Enrollment	9
Supplemental Table 5. Effects of Multivitamin/Multimineral Supplementation (vs. Placebo) on Incident Fracture by Tertile of Alternative Health Eating Index Score, According to Fracture Location	10
Supplemental Table 6. Effects of Multivitamin/Multimineral Supplementation (vs. Placebo) on Incident Fracture by Self-Rated General Health, According to Fracture Location	11

Supplemental Table 1. Composition of the COSMOS Centrum Silver Supplement

Vitamin or mineral (units)	Amount	% Daily Value (* Daily Value not established)
Biotin (μg)	30	10
Boron (μg)	-	-
Calcium (mg)	220	22
Chloride (mg)	72	2
Chromium (μg)	50	42
Copper (mg)	0.5	25
Folic Acid (μg)	400	100
Iodine (μg)	150	100
Iron (mg)	-	-
Lutein (μg)	250	*
Lycopene (μg)	300	*
Magnesium (mg)	50	13
Manganese (mg)	2.3	115
Molybdenum (μg)	45	60
Niacin (mg)	20	100
Nickel (μg)	5	*
Pantothenic Acid (mg)	10	100
Phosphorus (mg)	20	2
Potassium (mg)	80	2
Riboflavin (mg)	1.7	100
Selenium (μg)	19	27
Silicon (mg)	2	*
Thiamin (mg)	1.5	100
Vanadium (μg)	10	*
Vitamin A (IU)	2500<br>40% β-carotene	50
Vitamin B_{12} (μg)	25	417
Vitamin B_6 (mg)	3	150
Vitamin C (mg)	60	100
Vitamin D (IU)	1000	250
Vitamin E (IU)	50	167
Vitamin K (μg)	30	38
Zinc (mg)	11	73

Supplemental Table 2. Baseline characteristics of participants in the overall COSMOS cohort (21,442), according to randomized treatment assignment: Cocoa Extract Supplementation vs. Cocoa Extract Placebo

Variable	All	Cocoa Extract	Cocoa Extract Placebo
Demographic characteristics			
**Age group,** no. (%)	N=21,442		
60-69	9224 (43.0%)	4610 (43.0%)	4614 (43.0%)
70-74	5774 (26.9%)	2886 (26.9%)	2888 (26.9%)
75-79	3751 (17.5%)	1876 (17.5%)	1875 (17.5%)
80-84	1761 (8.2%)	882 (8.2%)	879 (8.2%)
≥85	932 (4.4%)	465 (4.3%)	467 (4.4%)
**Body Mass Index group,** no. (%), kg/m^2	N=21,018		
<25	7070 (33.64%)	3584 (34.12%)	3486 (33.16%)
25 to <30	8230 (39.16%)	4062 (38.67%)	4168 (39.65%)
≥30	5718 (27.21%)	2859 (27.22%)	2859 (27.19%)
Medical Conditions			
**Diabetes**	N=21,433		
Yes	2864 (13.36%)	1417 (13.22%)	1447 (13.50%)
**Multiple Sclerosis**	N=21,239		
Yes	66 (0.31%)	32 (0.30%)	34 (0.32%)
**Parkinson's Disease**	N=21,278		
Yes	81 (0.38%)	33 (0.31%)	48 (0.45%)
Medications (regular use)			
**Use of diabetes medication**	N=21,433		
Yes	2233 (10.42%)	1099 (10.26%)	1134 (10.58%)
**Use of selective serotonin reuptake inhibitors**	N=21,116		
Yes	1622 (7.68%)	830 (7.85%)	792 (7.51%)
**Use of proton pump inhibitors**	N=21,188		
Yes	2424 (11.44%)	1237 (11.66%)	1187 (11.22%)
**Use of corticosteroids**	N=20,937		
Yes	587 (2.80%)	310 (2.96%)	277 (2.65%)
**Use of bone loss/osteoporosis medications***	N=21,095		
Yes	1413 (6.70%)	720 (6.83%)	693 (6.57%)
**Use of thyroid hormone**	N=21,197		
Yes	3761 (17.74%)	1839 (17.35%)	1922 (18.14%)
Behavioral Characteristics			

Variable	All	Cocoa Extract	Cocoa Extract Placebo
**Sleep, hrs**	N=21,123		
<5	358 (1.69%)	175 (1.66%)	183 (1.73%)
5	1264 (5.98%)	638 (6.05%)	626 (5.92%)
6	4153 (19.66%)	2035 (19.28%)	2118 (20.04%)
7	8053 (38.12%)	4112 (38.97%)	3941 (37.28%)
8	5609 (26.55%)	2735 (25.92%)	2874 (27.19%)
9	1476 (6.99%)	749 (7.10%)	727 (6.88%)
≥10	210 (0.99%)	109 (1.03%)	101 (0.96%)
Physical Activity			
**Flights of stairs climbed daily**	N=21,019		
None	5124 (24.38%)	2533 (24.14%)	2591 (24.62%)
1-2 flights	4647 (22.11%)	2314 (22.05%)	2333 (22.17%)
3-4 flights	3414 (16.24%)	1703 (16.23%)	1711 (16.26%)
5-9 flights	4051 (19.27%)	2022 (19.27%)	2029 (19.28%)
10-14 flights	2334 (11.10%)	1190 (11.34%)	1144 (10.87%)
15 or more	1449 (6.89%)	732 (6.98%)	717 (6.81%)
**Usual walking pace**	N=21,166		
Don’t walk regularly	2381 (11.25%)	1161 (10.98%)	1220 (11.51%)
Easy (less than 2 mph)	3791 (17.91%)	1871 (17.70%)	1920 (18.12%)
Normal 2-2.9 mph	9585 (45.28%)	4792 (45.33%)	4793 (45.24%)
Brisk 3-3.9 mph	4919 (23.24%)	2489 (23.55%)	2430 (22.94%)
Very brisk 4 mph or faster	490 (2.32%)	258 (2.44%)	232 (2.19%)
**Physical Function limitations** in past 2 weeks			
**Climbing a flight of stairs without stopping** (N=21,007)			
Extremely	420 (2.00%)	214 (2.04%)	206 (1.96%)
Quite a bit	459 (2.18%)	226 (2.15%)	233 (2.22%)
Moderately	803 (3.82%)	399 (3.80%)	404 (3.85%)
Slightly	2138 (10.18%)	1048 (9.98%)	1090 (10.38%)
Not at all	17187 (81.82%)	8618 (82.04%)	8569 (81.59%)
**Dressing yourself** (N=21,103)			
Extremely	23 (0.11%)	10 (0.09%)	13 (0.12%)
Quite a bit	31 (0.15%)	15 (0.14%)	16 (0.15%)

Variable	All	Cocoa Extract	Cocoa Extract Placebo
Moderately	91 (0.43%)	44 (0.42%)	47 (0.45%)
Slightly	378 (1.79%)	183 (1.73%)	195 (1.85%)
Not at all	20580 (97.52%)	10299 (97.61%)	10281 (97.43%)
**Hurrying (as if to catch a bus) or jogging** (N=20,954)			
Extremely	913 (4.36%)	454 (4.33%)	459 (4.38%)
Quite a bit	856 (4.09%)	416 (3.97%)	440 (4.20%)
Moderately	1048 (5.00%)	532 (5.08%)	516 (4.93%)
Slightly	2574 (12.28%)	1312 (12.52%)	1262 (12.05%)
Not at all	15563 (74.27%)	7768 (74.11%)	7795 (74.44%)
**Showering and bathing** (N=21,111)			
Extremely	33 (0.16%)	16 (0.15%)	17 (0.16%)
Quite a bit	43 (0.20%)	20 (0.19%)	23 (0.22%)
Moderately	138 (0.65%)	71 (0.67%)	67 (0.63%)
Slightly	456 (2.16%)	219 (2.07%)	237 (2.25%)
Not at all	20441 (96.83%)	10229 (96.91%)	10212 (96.74%)
**Walking one block on level ground** (N=21,043)			
Extremely	239 (1.14%)	117 (1.11%)	122 (1.16%)
Quite a bit	293 (1.39%)	148 (1.41%)	145 (1.38%)
Moderately	478 (2.27%)	242 (2.30%)	236 (2.24%)
Slightly	1204 (5.72%)	585 (5.56%)	619 (5.88%)
Not at all	18829 (89.48%)	9425 (89.62%)	9404 (89.34%)
**Doing yard work, housework, or carrying groceries** (N=21,058)			
Extremely	248 (1.18%)	125 (1.19%)	123 (1.17%)
Quite a bit	419 (1.99%)	188 (1.78%)	231 (2.19%)
Moderately	941 (4.47%)	473 (4.49%)	468 (4.45%)
Slightly	2718 (12.91%)	1399 (13.28%)	1319 (12.53%)
Not at all	16732 (79.46%)	8348 (79.26%)	8384 (79.66%)
**General Health, Self-reported**	N=20,975		
Excellent	5633 (26.86%)	2859 (27.25%)	2774 (26.46%)
Very Good/Good	14709 (70.13%)	7321 (69.79%)	7388 (70.46%)
Fair/Poor	633 (3.02%)	310 (2.96%)	323 (3.08%)

Supplemental Table 3. Baseline characteristics of participants in the overall COSMOS cohort (21,442), according to randomized treatment assignment: Multivitamin/Multimineral Supplementation vs. Placebo

Variable	All[1]	Multivitamin/Multimineral Supplementation	Placebo
Demographic characteristics			
**Age group,** no. (%)			
60-69	9224 (43.0%)	4611 (43.0%)	4613 (43.0%)
70-74	5774 (27.0%)	2886 (26.9%)	2888 (26.9%)
75-79	3751 (17.5%)	1875 (17.5%)	1876 (17.5%)
80-84	1761 (8.2%)	889 (8.3%)	872 (8.1%)
≥85	932 (4.4%)	459 (4.3%)	473 (4.4%)
**Body Mass Index group,** no. (%), kg/m^2			
<25	7070 (33.6%)	3533 (33.7%)	3537 (33.6%)
25 to <30	8230 (39.16%)	4166 (39.7%)	4064 (38.6%)
≥30	5718 (27.21%)	2800 (26.7%)	2918 (27.7%)
Medical Conditions			
**Diabetes**	N=21433		
Yes	2864 (13.4%)	1415 (13.2%)	1449 (13.5%)
**Multiple Sclerosis**	N=21239		
Yes	66 (0.3%)	38 (0.4%)	28 (0.3%)
**Parkinson's Disease**	N=21278		
Yes	81 (0.4%)	32 (0.3%)	49 (0.5%)
Medications (regular use)			
**Use of diabetes medication**	N=21433		
Yes	2233 (10.4%)	1098 (10.3%)	1135 (10.6%)
**Use of selective serotonin reuptake inhibitors**	N=21,116		
Yes	1622 (7.7%)	767 (7.3%)	855 (8.1%)
**Use of proton pump inhibitors**	N=21,188		
Yes	2424 (11.4%)	1178 (11.1%)	1246 (11.8%)
**Use of corticosteroids**	N=20,937		
Yes	587 (2.8%)	265 (2.5%)	322 (3.1%)

[1] Information was lacking regarding ethnicity in 863 participants, body mass index in 424 participants, and leisure time physical activity in 258 participants.

Variable	All[1]	Multivitamin/Multimineral Supplementation	Placebo
**Use of prescription osteoporosis medications[2]**	N=21,095		
Yes	1413 (6.7%)	685 (6.5%)	728 (6.9%)
**Use of thyroid hormone**	N=21,197		
Yes	3761 (17.7%)	1881 (17.8%)	1880 (17.7%)
Behavioral Characteristics			
**Sleep, hrs**	N=21123		
<5	358 (1.7%)	181 (1.7%)	177 (1.7%)
5	1264 (6.0%)	647 (6.1%)	617 (5.9%)
6	4153 (19.7%)	2073 (19.6%)	2080 (19.7%)
7	8053 (38.1%)	3991 (37.8%)	4062 (38.5%)
8	5609 (26.6%)	2825 (26.7%)	2784 (26.4%)
9	1476 (7.0%)	750 (7.1%)	726 (6.9%)
≥10	210 (1.0%)	105 (1.0%)	105 (1.0%)
Physical Activity			
**Flights of stairs climbed daily**	N=21019		
None	5124 (24.4%)	2558 (24.3%)	2566 (24.4%)
1-2 flights	4647 (22.1%)	2307 (21.9%)	2340 (22.3%)
3-4 flights	3414 (16.2%)	1715 (16.3%)	1699 (16.2%)
5-9 flights	4051 (19.3%)	2020 (19.2%)	2031 (19.3%)
10-14 flights	2334 (11.1%)	1185 (11.3%)	1149 (10.9%)
15 or more	1449 (6.9%)	735 (7.0%)	714 (6.8%)
**Usual walking pace**	N=21166		
Don't walk regularly	2381 (11.3%)	1193 (11.3%)	1188 (11.2%)
Easy (less than 2 mph)	3791 (18.0%)	1901 (18.0%)	1890 (17.9%)
Normal 2-2.9 mph	9585 (45.3%)	4771 (45.1%)	4814 (45.5%)
Brisk 3-3.9 mph	4919 (23.2%)	2484 (23.5%)	2435 (23.0%)
Very brisk 4 mph or faster	490 (2.3%)	238 (2.3%)	252 (2.4%)
**Physical Function limitations** in past 2 weeks			
**Climbing a flight of stairs without stopping**	N=21007		
Extremely	420 (2.0%)	215 (2.1%)	205 (2.0%)

[2] alendronate, risedronate, ibandronate, zoledronic acid, denosumab, teriparatide, raloxifene, or calcitonin

Variable	All[1]	Multivitamin/Multimineral Supplementation	Placebo
Quite a bit	459 (2.2%)	234 (2.2%)	225 (2.1%)
Moderately	803 (3.8%)	390 (3.7%)	413 (3.9%)
Slightly	2138 (10.2%)	1037 (9.9%)	1101 (10.5%)
Not at all	17187 (81.8%)	8623 (82.1%)	8564 (81.5%)
**Dressing yourself**	(N=21,103)		
Extremely	23 (0.1%)	16 (0.2%)	7 (0.1%)
Quite a bit	31 (0.2%)	18 (0.2%)	13 (0.1%)
Moderately	91 (0.4%)	44 (0.4%)	47 (0.5%)
Slightly	378 (1.8%)	191 (1.8%)	187 (1.8%)
Not at all	20580 (97.5%)	10284 (97.5%)	10296 (97.6%)
**Hurrying (as if to catch a bus) or jogging**	(N=20,954)		
Extremely	913 (4.4%)	459 (4.4%)	454 (4.3%)
Quite a bit	856 (4.1%)	432 (4.1%)	424 (4.1%)
Moderately	1048 (5.0%)	499 (4.8%)	549 (5.2%)
Slightly	2574 (12.3%)	1252 (12.0%)	1322 (12.6%)
Not at all	15563 (74.3%)	7837 (74.8%)	7726 (73.8%)
**Showering and bathing**	(N=21,111)		
Extremely	33 (0.2%)	22 (0.2%)	11 (0.1%)
Quite a bit	43 (0.2%)	24 (0.2%)	19 (0.2%)
Moderately	138 (0.7%)	71 (0.7%)	67 (0.6%)
Slightly	456 (2.2%)	224 (2.1%)	232 (2.2%)
Not at all	20441 (96.8%)	10218 (96.8%)	10223 (96.9%)
**Walking one block on level ground**	(N=21,043)		
Extremely	239 (1.1%)	124 (1.2%)	115 (1.1%)
Quite a bit	293 (1.4%)	152 (1.5%)	141 (1.3%)
Moderately	478 (2.3%)	239 (2.3%)	239 (2.3%)
Slightly	1204 (5.7%)	573 (5.5%)	631 (6.0%)
Not at all	18829 (89.5%)	9429 (89.7%)	9400 (89.3%)
**Doing yard work, housework, or carrying groceries**	(N=21,058)		
Extremely	248 (1.2%)	129 (1.2%)	119 (1.1%)
Quite a bit	419 (2.0%)	217 (2.1%)	202 (1.9%)
Moderately	941 (4.5%)	466 (4.4%)	475 (4.5%)
Slightly	2718 (12.9%)	1320 (12.5%)	1398 (13.3%)
Not at all	16732 (79.5%)	8399 (79.8%)	8333 (79.2%)

Supplemental Table 4. Locations of Prevalent Fractures Prior to Trial Enrollment

Fracture location	Cocoa extract group (n = 10,719)	Cocoa extract placebo group (n = 10,723)	Multivitamin/Multimineral (MVM) Group (10,720)	MVM Placebo group (n = 10,722)
	n (%)	n (%)	n (%)	n (%)
Hip	135 (6.3)	116 (5.5%)	124 (6.0%)	127 (5.8%)
Clinical spine	111 (5.2%)	82 (3.9%)	83 (4.0%)	110 (5.1%)
Lower arm	412 (19.1%)	409 (19.5%)	392 (18.9%)	429 (19.7%)
Upper arm	185 (8.6%)	180 (8.6%)	192 (9.3%)	173 (8.0%)
Lower leg	277 (12.9%)	310 (14.8%)	298 (14.4%)	289 (13.3%)
Upper leg	60 (2.8%)	78 (3.7%)	68 (3.3%)	70 (3.2%)
Foot	582 (27.0%)	579 (27.7%)	559 (27.0%)	602 (27.7%)
Other	861 (40.0%)	803 (38.4)	825 (39.8%)	839 (38.6%)

Supplemental Table 5. Effects of Multivitamin/Multimineral Supplementation (vs. Placebo) on Incident Fracture by Tertile of Alternative Healthy Eating Index Score, According to Fracture Location

		# Events	HR (95% CI)
**Clinical**			
	Lowest tertile	561	0.99 (0.84-1.17)
	Middle tertile	629	1.02 (0.87-1.20)
	Highest tertile	660	1.27 (1.09-1.48)
**Hip**			
	Lowest tertile	52	0.85 (0.49-1.48)
	Middle tertile	59	1.05 (0.63-1.75)
	Highest tertile	57	1.61 (0.94-2.76)
**Nonvertebral**			
	Lowest tertile	528	1.01 (0.85-1.20)
	Middle tertile	581	1.01 (0.85-1.18)
	Highest tertile	624	1.27 (1.08-1.49)
**Clinical Spine**			
	Lowest tertile	40	0.89 (0.48-1.67)
	Middle tertile	57	1.13 (0.67-1.90)
	Highest tertile	51	1.45 (0.82-2.54)
**Forearm/wrist**			
	Lowest tertile	90	0.92 (0.60-1.39)
	Middle tertile	106	0.87 (0.59-1.27)
	Highest tertile	107	1.41 (0.96-2.07)
**Major osteoporotic**			
	Lowest tertile	229	0.90 (0.69-1.16)
	Middle tertile	282	1.02 (0.81-1.29)
	Highest tertile	279	1.41 (1.11-1.78)
**Pelvic**			
	Lowest tertile	20	0.43 (0.16-1.11)
	Middle tertile	29	0.61 (0.29-1.29)
	Highest tertile	34	1.06 (0.54-2.08)

Supplemental Table 6. Effects of Multivitamin/Multimineral Supplementation (vs. Placebo) on Incident Fracture by Self-Rated General Health, According to Fracture Location

			**Hazard Ratio (95% Confidence Interval)**
**Clinical**			
	Excellent	462	0.95 (0.80-1.15)
	Very good/good	1482	1.13 (1.02-1.25)
	Fair/poor	78	1.66 (1.04-2.65)
**Hip**			
	Excellent	34	0.85 (0.44-1.69)
	Very good/good	133	1.06 (0.76-1.49)
	Fair/poor	11	2.80 (0.72-10.87)
**Nonvertebral**			
	Excellent	431	0.96 (0.79-1.16)
	Very good/good	1393	1.13 (1.02-1.26)
	Fair/poor	70	1.72 (1.06-2.84)
**Clinical Spine**			
	Excellent	29	1.03 (0.50-2.13)
	Very good/good	118	1.30 (0.90-1.87)
	Fair/poor	13	1.85 (0.57-6.02)
**Forearm/wrist**			
	Excellent	78	1.09 (0.70-1.70)
	Very good/good	237	1.06 (0.82-1.37)
	Fair/poor	9	0.93 (0.25-3.55)
**Major osteoporotic**			
	Excellent	186	1.01 (0.76-1.35)
	Very good/good	629	1.11 (0.95-1.30)
	Fair/poor	39	1.87 (0.96-3.65)
**Pelvic**			
	Excellent	22	0.87 (0.38-2.03)
	Very good/good	63	0.66 (0.40-1.09)
	Fair/poor	4	--[3]

[3] Number of cases inadequate for reliable estimation of hazard ratio.
